# The role of specialist palliative care in individuals “living beyond cancer”: a narrative review of the literature

**DOI:** 10.1007/s00520-024-08598-w

**Published:** 2024-06-06

**Authors:** Amy Taylor, Andrew Davies

**Affiliations:** 1 Research Fellow / Specialty Trainee in Palliative Medicine, Trinity College Dublin and Our Lady’s Hospice, Dublin, Ireland; 2 Palliative Medicine, Trinity College Dublin and University College Dublin and Our Lady’s Hospice, Dublin, Ireland; 3 Education & Research Centre, Our Lady’s Hospice Dublin, Harold’s Cross, Dublin, D6W RY72 Ireland

**Keywords:** Palliative care, Cancer survivors, Cancer survivorship, Living beyond cancer

## Abstract

**Purpose:**

Many patients living beyond cancer experience significant unmet needs, although few of these patients are currently reviewed by specialist palliative care teams (SPCTs). The aim of this narrative review was to explore the current and potential role of SPCTs in this cohort of patients.

**Methods:**

A search strategy was developed for Medline, and adapted for Embase, CINAHL, and PsycInfo. Additionally, websites of leading oncology, cancer survivorship, and specialist palliative care organisations were examined. The focus of the search was on individuals living beyond cancer rather than other groups of cancer survivors.

**Results:**

111 articles were retrieved from the search for full text review, and 101 other sources of information were identified after hand searching the reference lists of the full text articles, and the aforesaid websites. The themes of the review encompass the definition of palliative care/specialist palliative care, current models of specialist palliative care, core activities of SPCTs, relevant expertise of SPCTs, and potential barriers to change in relation to extending their support and expertise to individuals living beyond cancer. The review identified a paucity of evidence to support the role of SPCTs in the management of patients living beyond cancer.

**Conclusions:**

Individuals living beyond cancer have many unmet needs, and specific services are required to manage these problems. Currently, there is limited evidence to support the role of specialist palliative care teams in the management of this cohort of people, and several potential barriers to greater involvement, including limited resources, and lack of relevant expertise.

## Introduction

“Cancer survivor” is a ubiquitous term, which has various connotations amongst healthcare professionals, patients with cancer, family carers, and the general population [1]. The National Cancer Institute (NCI) in the USA states that “an individual is considered a cancer survivor from the time of diagnosis through the balance of life. There are many types of survivors, including those living with cancer and those free of cancer” [2]. The NCI definition is widely quoted, and has been adopted to a greater or lesser extent by many other national and international organisations. However, the colloquial (general population) meaning of a survivor is “one remaining alive after some disaster in which others perish”, i.e. a cancer survivor is an individual that has been “cured” of cancer [3]. This disparity in meaning is highlighted by studies investigating the acceptability of the term cancer survivor amongst different cohorts of patients with cancer [4].

**Fig. 1:**
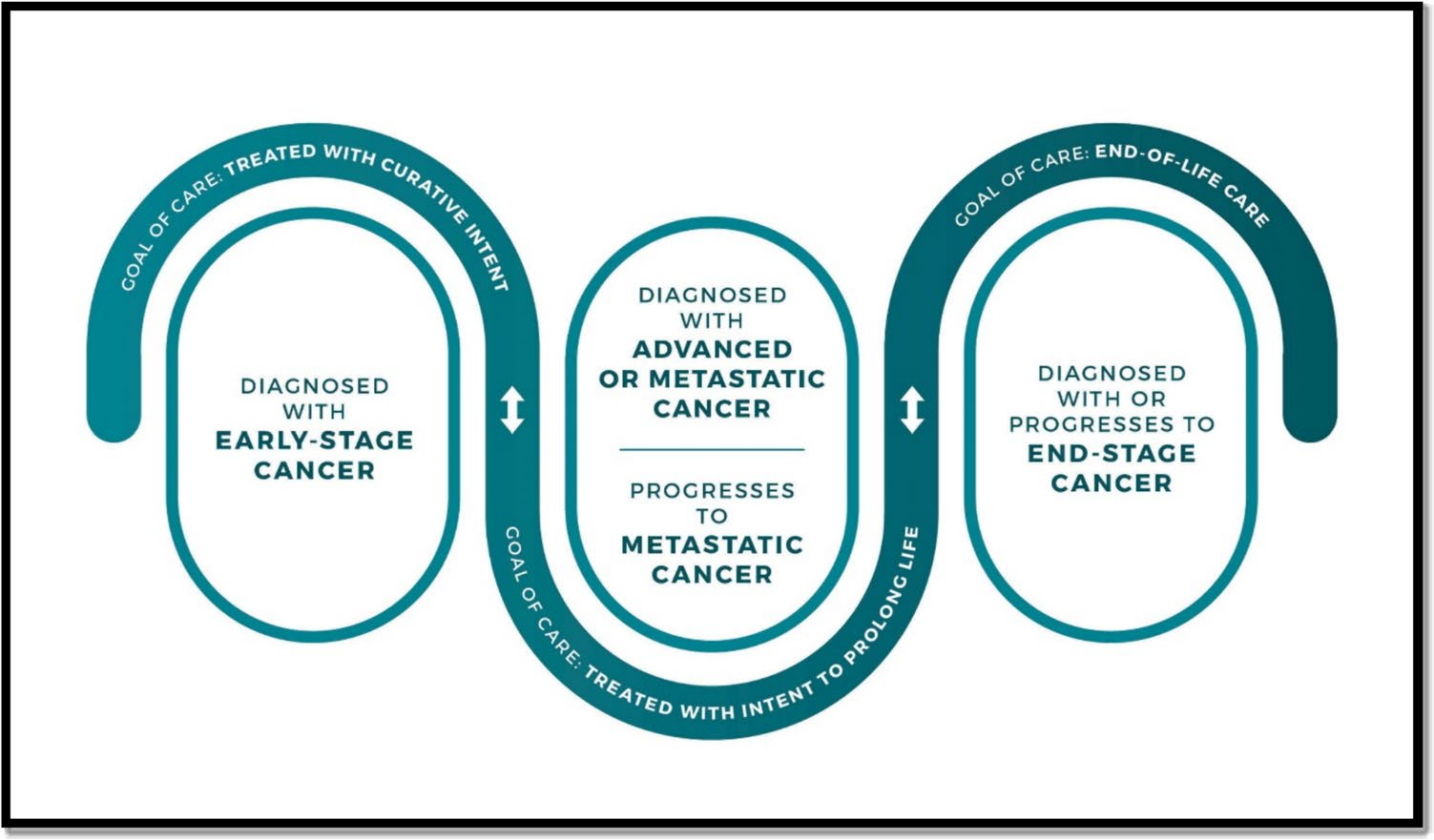
National Cancer Institute phases of cancer survivorship [2]

The NCI acknowledges this disparity, and notes that “years ago, some suggested that the definition should only include people who were cancer-free for a minimum amount of time after their diagnosis”, and goes on to say that “this term (*cancer survivor*) is meant to capture a population of those with a history of cancer rather than to provide a label that may or may not resonate with individuals” [2]. The NCI definition of cancer survivor is supported by a three phase model of cancer survivorship (Fig. 1), which specifically includes patients with “end stage cancer”, but surprisingly not individuals that have been cured of cancer [2]. In response to concerns about nomenclature, the terms “living with cancer”, “living through cancer”, and “living beyond cancer” (either alone or together, e.g. “living with and beyond cancer”) have been introduced [5]: living beyond cancer “refers to post-treatment and long-term survivorship” (i.e. individuals that have been potentially cured of cancer) [6].

Many cancer survivors experience significant unmet needs, which encompass the continuum of physical, psychological, spiritual, and social domains (Table 1) [7], and which relate to the cancer (directly or indirectly), the cancer treatment, and/or the presence of a chronic (potentially life-limiting) disease [8]. Indeed, these unmet needs are the primary driver for the ongoing discussions around greater involvement of specialist palliative care teams (SPCTs) in the management of individuals living beyond cancer, and similarly underserved groups of cancer survivors, e.g. cancer patients with stable disease on maintenance anticancer treatment. Importantly, there is significant heterogeneity amongst individuals in the same phase, and especially between individuals in different phases, in terms of the problems experienced, and the most suitable options for management [9]. Hence, healthcare services for cancer survivors need to be tailored to the different phases / subgroups of patients (and then personalised to the individual patient).

**Table 1:** Unmet needs of cancer survivors (adapted from Burg et al., 2015) [7]

Unmet domain	Description	% individuals reporting
Physical	Needs and issues experienced in or affecting the body, including pain, symptoms, sexual dysfunction, and care of body (such as diet, exercise, and rest)	38.2%
Financial	Needs related to money, insurance, and the affordability of needed services and products	20.3%
Education / information	Needs related to unanswered questions and the lack of knowledge regarding what to expect as a cancer survivor, follow-up care, self-care, cancer and health research, and cancer risks, causes, and prevention	19.5%
Personal control	Needs related to an individual’s ability to maintain autonomy in terms of the physical self (sexual function, evacuation, and ambulation) and the social self (disclosure about cancer and ability to make plans and socialize). Also includes wishes to return to “normal” and finding a “new normal”	16.4%
System of care	Needs related to the health care system, including constraints and flaws that affect early detection, diagnosis, treatment, follow-up care, continuity of care, and inadequate response from health care providers	15.5%
Resources	Needs related to availability and access to supplies, equipment, therapies and medications (including alternative and complementary), and transportation services	13.8%
Emotions / mental health	Needs related to psychological issues, including fear (recurrence, new cancers, death, and dying), depression, anxiety, and negative feelings (mistrust toward body, anger, and guilt)	13.7%
Social support	Needs related to psychosocial and interpersonal issues, including intimacy, access to support groups, opportunities to use one’s own experiences to help others, and participation in social situations	12.7%
Societal	Needs revealed from respondents’ commentary about conditions and issues related to society’s response to cancer, including social norms, discrimination, misinformation, policies, and resource allocation (insurance coverage)	10.0%
Communication	Needs related to discourse (talking) and information exchange (explaining) about cancer and cancer experience with others (including survivor and doctor and survivor and family/friends/employers) and among medical providers	8.5%
Provider relationship	Needs related to trust in health care providers, including decision-making, follow-through, follow-up, and support	8.5%
Cure	Needs related to a wish for a cure for cancer and hopes of effective treatments for self and others	3.5%
Body image	Needs related to negative perception of body, including feeling unattractive and/or ashamed and loss of trust in body	3.5%
Survivor identity	Pertains to the respondent either explicitly identifying or not identifying as a cancer survivor because the respondent does not like the term “survivor” or feels that he or she has not reached a specific milestone to be called a survivor (eg, not still in treatment or living a specific number of years since the diagnosis)	3.1%
Employment	Needs pertaining to maintaining or obtaining a source of income that is appropriate given the cancer experience	2.3%
Existential	Needs pertaining to attaining peace in life and spirituality and making sense or meaning of the cancer experience	0.6%

The aim of this narrative review article is to explore the potential for greater involvement of SPCTs in supporting individuals living beyond cancer as already defined [6], rather than other cohorts of “cancer survivors”: the objectives are to define palliative care (and specialist palliative care), to outline current models of specialist palliative care, to outline the core activities of SPCTs, to determine the relevant expertise of SPCTs, and to highlight the potential barriers to change.

## Methodology

A literature search was initially undertaken in May 2023, and subsequently updated in January 2024. The search included four electronic databases (Medline, Embase, CINAHL, PsycInfo), the internet (i.e. websites of leading oncology, cancer survivorship, and specialist palliative care organisations), and hand searching of specialist palliative care textbooks. A broad search strategy was developed for Medline (Appendix 1), and adapted as needed for the other electronic databases: the electronic databases were searched from their inception to the present. Basically, we were seeking articles relating to the role / potential role of specialist palliative care teams in the management of cancer survivors (and specifically individuals living beyond cancer). All types of articles were considered, including editorials / commentaries, review articles, and particularly original research (including conference abstracts). Articles relating to children were not reviewed. Non-English articles were not reviewed, unless there was an English abstract (which was reviewed).

EndNote 20™ bibliographic software (Clarivate Analytics LLP, USA) was used to store the retrieved articles, whilst Covidence systematic review software (Veritas Health Innovation, Australia) was used to screen these retrieved articles. The two authors independently screened the titles and abstracts for full text articles to review. Disagreements were resolved by consensus. The two authors also independently reviewed the full text articles, and extracted the relevant information using a review-specific template: this information was predetermined, and was collated using a PICOS framework (i.e. Population, Intervention, Comparison, Outcome, Study Design). The reference lists of all retrieved full text articles were reviewed for further potential articles or sources of information.

## Results

**Fig. 2:**
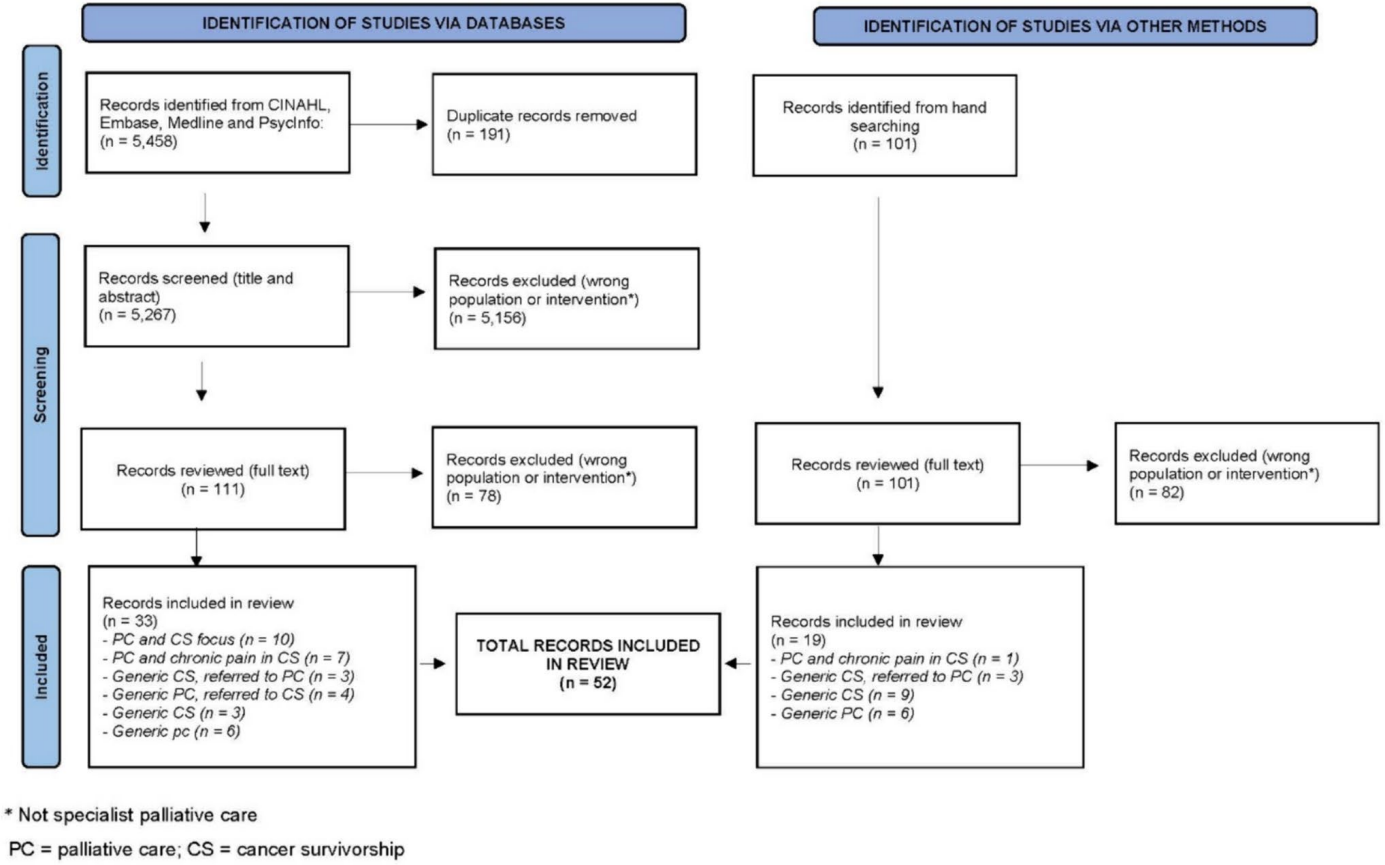
Search of the literature

The searches of the literature identified 5458 articles, which included 191 duplicate articles that were removed, and 5156 “irrelevant” articles that were excluded (on the basis of their titles and abstracts). Thus, 111 articles from the search underwent full text evaluation. Another 101 articles / sources of information were identified (and underwent full text evaluation) following searching the internet, hand searching of specialist palliative care textbooks, and hand searching of the reference lists of the included full text articles. Figure 2 gives an overview of the search of the literature.

Ten articles had a specific focus on palliative care and cancer survivorship [9–18]: five were commentaries [9, 10, 13, 15, 16], twowere literature reviews [11, 18], one was an editorial [14], one was a letter to the editor [12], and one involved original research (i.e. postal survey) [17]. Of note, six of these articles were published in oncology journals (three in oncology nursing journals) [10, 11, 13–15, 18], two in generic journals [9, 16], and only two in palliative care journals [12, 17].

A further eight articles concerned chronic pain in cancer survivors, and the actual / potential role of SPCTs in managing this cohort of patients [19–26]. Six of the remaining references were either generic cancer survivorship articles (that mentioned palliative care) [27–31], and four were generic palliative care articles (that mentioned cancer survivors / survivorship) [32–35]. Again, few of these articles were published in mainstream palliative care journals.

## Literature review

### Definition of “palliative care”

A number of definitions have been proposed for palliative care [36], although the most widely quoted ones are the older World Health Organization (WHO) definitions [37, 38], and the newer International Association for Hospice and Palliative Care (IAHPC) definition (which evolved from the later WHO definition) [39]. The IAHPC define palliative care as “the active holistic care of individuals across all ages with serious health-related suffering due to severe illness and especially of those near the end of life. It aims to improve the quality of life of patients, their families and their caregivers” [39]. The definition is supported by a series of additional characteristics, which include “is applicable throughout the course of an illness, according to the patient’s needs”, and “is provided in conjunction with disease-modifying therapies whenever needed” [39].

### Definition of “specialist palliative care”

The European Association of Palliative Care (EAPC) define three “levels” of palliative care: a) palliative care approach – “ applies to those with limited experience and knowledge in dealing with palliative care but can apply the basic principles of good palliative care”; b) generalist palliative care – “applies to those who are frequently involved with palliative care and have some specialist palliative care knowledge”; and c) specialist palliative care – “team members must be highly qualified and should have their main focus of work in palliative care” [40].

It is important to note that most palliative care is currently provided by generalists (e.g. general practitioners, oncology clinicians), rather than palliative care specialists due to resource constraints, and that this scenario is unlikely to change due to ongoing / increasing workforce challenges (i.e. recruitment, retention / natural turnover) [41], changes in population demographics (i.e. higher population, more aged population) [42], and improvements in oncology treatments / outcomes (resulting more patients living through cancer, and more patients living beyond cancer).

### Current models of specialist palliative care

**Fig. 3:**
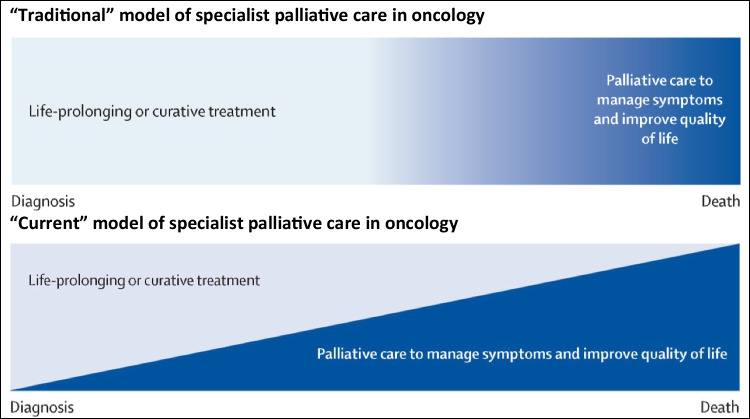
Traditional and current model of specialist palliative care in oncology [adapted from 32]

The “traditional” model of specialist palliative care focussed on patients with advanced cancer, and involved both symptom control and end-of-life care (Fig. 3) [32]. The “current” model of specialist palliative care reiterates the relevance of end-of-life care, but also highlights the importance of symptom control in other groups of patients with cancer (from the point of diagnosis) [32]. Moreover, the American Society of Clinical Oncology (ASCO) recommend that “patients with advanced cancer, whether inpatient or outpatient, should receive dedicated palliativecare services early in the disease course, concurrent with active treatment” [43]. The latter is often referred to as “early” palliative care, and is endorsed by other national and international oncology organisations [44].

The current model of specialist palliative care is from the Lancet Oncology Commission on Integration of Oncology and Palliative Care [32]. As can be seen, the model does not address the issue of individuals living beyond cancer, and the Commission suggests that cancer survivorship (meaning living beyond cancer) falls under the remit of supportive care rather than palliative care. The latter is backed up by the Multinational Association of Supportive Care in Cancer (MASCC), who define supportive care as “the prevention and management of the adverse effects of cancer and its treatment. This includes management of physical and psychological symptoms and side effects across the continuum of the cancer journey from diagnosis through treatment to post-treatment care. Supportive care aims to improve the quality of rehabilitation, secondary cancer prevention, survivorship, and end-of-life care” [45].

Supportive care has been used by some as a substitute for palliative care, with a major driver for this change being the widely held opinion that palliative care is synonymous with end-of-life care. However, although palliative care is an integral component of supportive care (as previously defined), supportive care is much more than palliative care, and requires input from a range of specialist teams and services [33]. Importantly, most SPCTs often have limited knowledge / experience of managing the active toxicities of anticancer treatments, and especially the newer immunotherapies and targeted treatments [34]. Furthermore, most SPCTs have even more limited knowledge / experience of managing the chronic toxicities of anticancer treatments, which has major implications for their involvement in the care of individuals living beyond cancer (see below).

Significantly, according to the NCI definition [2], all patients with a history of cancer are cancer survivors, including those with advanced / progressive disease, and even those in the final (terminal) stage of their illness. Thus, SPCTs already manage significant number of “cancer survivors”, although as previously indicated there is usually a clear demarcation between those subgroups that are routinely reviewed by these services, and those subgroups that are rarely (if ever) reviewed by these services (Table 2). Currently, SPCTs are seldom involved in the management of individuals living beyond cancer.

**Table 2:** Subgroups of “cancer survivors” and typical engagement with specialist palliative care teams

LIVING WITH CANCER (patient has active disease)	
Pre-oncology treatment (primary)	Hospital-based SPCTs may be involved in cancer symptom control
On oncology treatment (primary or other)—“curative” pathway	Hospital-based SPCTs often involved in cancer symptom control
On oncology treatment (primary or other)—“palliative” pathway	Hospital-based SPCTs often involved in cancer symptom control (and other core functions of specialist palliative care – see Box 1). Patients with advanced disease may also be referred to community based SPCTs
Not on oncology treatment—“watch and wait” pathway	Hospital-based SPCTs may be involved in cancer symptom control
Not on oncology treatment—no further treatment planned (advanced disease)	Patients usually supported by community-based SPCTs
LIVING THROUGH / BEYOND CANCER (patient has inactive disease)	
On active oncology follow up (some risk of recurrence)	Generally not reviewed by SPCTs
Not on active oncology follow up (low risk of recurrence – potentially “cured”)	Generally not reviewed by SPCTs

### Core activities of specialist palliative care teams

SPCTs are extremely heterogenous in terms of their membership, services provided, and populations served [34]. Nevertheless, most SPCTs provide the core functions outlined in Box 1, although this range of services is usually restricted to patients with advanced cancer. As discussed, some SPCTs also provide early palliative care, some provide supportive care, and a smaller number also provide services for individuals living beyond cancer (generally symptom control).

### Relevant expertise of specialist palliative care teams

The influential Institute of Medicine and National Research Council of the National Academies report outlined four “essential components of survivorship care” [27]: 1) prevention of recurrent and new cancers, and of other late effects; 2) surveillance for cancer spread, recurrence, or second cancers; assessment of medical and psychosocial late effects; 3) intervention for consequences of cancer and its treatment, for example: medical problems such as lymphedema and sexual dysfunction; symptoms, including pain and fatigue; psychological distress experienced by cancer survivors and their caregivers; and concerns related to employment, insurance, and disability; and 4) coordination between specialists and primary care providers to ensure that all of the survivor’s health needs are met.

**Box 1:**
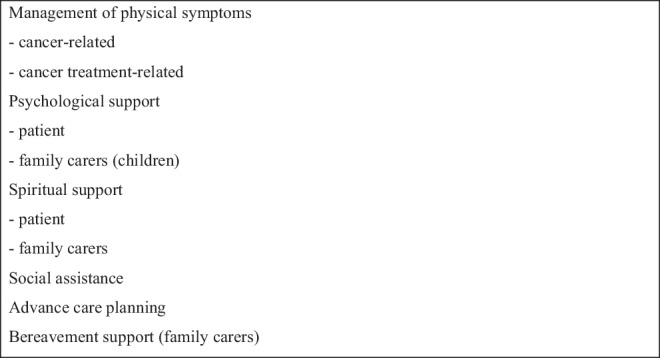
“Core” activities of specialist palliative care teams

At first glance, there seem to be many synergies between the core activities of SPCTs, the essential components of survivorship care [27], and the well described unmet physical and psychosocial needs of individuals living beyond cancer (Table 1) [7, 46]. Unsurprisingly, there is some (limited) data to suggest support for the involvement of SPCTs in “long term cancer survivors” from oncologists, general practitioners, and oncology patients themselves [28]. Moreover, a number of oncology-related organisations have recommended involvement of SPCTs in the management of cancer survivors (and specifically the treatment of pain and related problems) [29, 30, 31]. However, there is less obvious support from palliative care-related organisations [47]. The reasons for the apparent reticence are discussed in the following section.

Many of the focused (palliative care and cancer survivorship) articles supported a palliative care approach to the management of individuals living beyond cancer, which is unsurprising since a palliative care approach is essentially a holistic approach (and is the basis for “good” healthcare irrespective of the cohort of patients) [9, 16]. However, some of the focused articles supported actual involvement of SPCTs, and this ranged from targeted interventions in specific cohorts (especially symptom control) [9, 11], to provision of more wide-ranging activities in those with unmet needs (e.g. advance care planning, coordination of care) [10, 13].

In terms of pain, SPCTs are adept in the treatment of acute / chronic cancer-related pain, and to a lesser extent acute cancer treatment-related pain. However, they are generally inexperienced in the treatment of chronic cancer treatment-related pain, which is a major problem in individuals living beyond cancer [48]. Moreover, the treatment of chronic cancer-treatment-related pain is fundamentally different to the treatment of acute / chronic cancer-related pain (especially in individuals with a limited prognosis) [31]. Thus, the neurophysiology is different, the efficacy of analgesics is often different (usually decreased), the risk / benefit of analgesics is often different (usually increased), and the focus of care is on improving patient function [19, 49]. Additionally, non-pharmacological interventions (e.g. physical exercise, psychological interventions) have a major role in the management of chronic pain, and these interventions are invariably provided by multidisciplinary chronic pain teams rather than SPCTs.

The same is equally true for the treatment of other physical symptoms, and to a lesser extent psychological symptoms. For instance, many of the chronic gastrointestinal problems caused by anticancer treatments require specialist investigations, and/or specialist interventions (which are outside the scope of practice of SPCTs) [50]: an example would be rectal bleeding from radiation-induced telangiectasia following pelvic radiotherapy. Similarly, SPCTs are not trained to deal with fear of cancer progression [9], which is a common problem amongst cancer survivors. Moreover, SPCTs do not tend to have experience in other “essential components of survivorship care” such as cancer surveillance, and cancer prevention [27], or the certain other unmet needs in this cohort of patients (e.g. financial toxicity, issues relating to employment) (Table 1) [7].

Importantly, there is currently a paucity of evidence to support the role of SPCTs in the management of patients living beyond cancer [18].

### Potential barriers to change

There are a number of barriers to greater specialist palliative care involvement in individuals living beyond cancer (and similarly underserved groups of cancer survivors):

- ***Limited resources***

The National Coalition for Hospice and Palliative Care (NCHPC) in the USA contend that SPCTs do not have the capacity to see individuals living beyond cancer (“long-term cancer survivors”) as well as their usual cohorts of patients [47]. Moreover, this situation is likely to worsen due to changes in expected population demographics (resulting in greater numbers of patients, and smaller numbers of healthcare workers) [41]. However, the NCHPC suggest that SPCTs may be able to support dedicated cancer survivorship services in terms of education about the principles of palliative care, development of treatment algorithms, and potentially sharing relevant resources [47].

Importantly, any increase in service provision from SPCTs will require dedicated funding [13], and this will necessitate either provision of additional funding, or reallocation of current funding (which may have negative impacts on existing services). Moreover, this funding would need to be ongoing, and so necessarily supported by changes to healthcare policy / priorities at regional and national levels [18].

- ***Lack of relevant expertise***

As discussed, many of the problems encountered by individuals living beyond cancer are fundamentally different from those encountered in patients with advanced cancer (Table 1), and so SPCTs will need additional education / training if they are to provide the most suitable options for individuals living beyond cancer [35]. Indeed, extrapolating practice from patients with advanced cancer to individuals living beyond cancer is likely to lead to utilisation of ineffective interventions, and potentially to development of significant complications.

- ***Nomenclature (“palliative care”)***

The view that palliative care is synonymous with end-of-life care is commonplace amongst healthcare professionals, patients (including cancer survivors) [17], family carers, and the general population [13]. Indeed, such views have resulted in a reluctance from some oncology professionals (and other healthcare professionals) to refer patients with non-advanced cancer to SPCTs [51]. Thus, consideration will need to be given to improving understanding about palliative care, and/or rebranding of relevant services (e.g. “supportive and palliative care team”).

- ***Lack of professional willingness***

Many SPCTs have opted for a variety of reasons to solely manage patients with advanced cancer (and other life-limiting diseases), and have not engaged in developments such as early palliative care, and/or supportive care for cancer patients [34]. It is likely that the same would be true about providing services for individuals living beyond cancer.

## Strengths / limitations

This review is unique in terms of its focus, and its strength relates to the methods utilised, whilst its weakness (limitations) relates to the current paucity of evidence regarding the involvement of specialist palliative care teams in the management of individuals living beyond cancer, i.e. small number of studies, limited quality of studies. Indeed, this paucity of evidence lends itself to performing a narrative review, rather than other types of review (e.g. scoping, systematic).

## Conclusion

Individuals living beyond cancer have many unmet needs, and specific services are required to manage these problems. Moreover, a whole system, multidisciplinary, and inter-specialty approach is needed given the scale and breadth of these problems. Specialist palliative care teams could have a role to play, but this would require targeted education / training, and especially research to ensure that this input was effective and well tolerated (and did not disadvantage the usual cohort of palliative care patients).

## Data Availability

No datasets were generated or analysed during the current study.

## Appendix 1

Medline search strategy

1. Cancer survivors MESH term
2. Cancer survivorship key word
3. Cancer survivor key word
4. Living with cancer key word
5. Living beyond cancer key word
6. 1 or 2–5
7. Survivors MESH term
8. Survivorship MESH term
9. Survivor key word
10. 7 or 8–9
11. Neoplasms MESH term
12. Cancer key word
13. 11 or 12
14. 10 and 13
15. 6 or 14
16. Palliative care MESH term
17. Terminal care MESH term
18. End of life care key word
19. Hospice care MESH term
20. 16 or 17–19
21. 15 and 20
